# Health outcomes and experiences of direct-to-consumer high-intensity screening using both whole-body magnetic resonance imaging and cardiological examination

**DOI:** 10.1371/journal.pone.0242066

**Published:** 2020-11-20

**Authors:** Daniel Hommes, Derk Klatte, Wilma Otten, Maaike Beltman, Günter Klass, Aria Zand, René Sprangers

**Affiliations:** 1 Dept. of Gastroenterology, Leiden University Medical Centre, Leiden, The Netherlands; 2 Dept. of Internal Medicine, University of California Los Angeles (UCLA), Los Angeles, California, United States of America; 3 Expertise Group Child Health, Unit Healthy Living, The Netherlands Organization for Applied Scientific Research (TNO), The Netherlands; 4 Dept. of Radiology, Mathias-Spital, Rheine, Germany; 5 Prescan Inc., Hengelo, The Netherlands; McLean Hospital, UNITED STATES

## Abstract

**Background:**

Alongside a clinical and research setting, whole body magnetic resonance imaging (WB-MRI) is increasingly offered as a direct-to-consumer screening service. Data is needed on the clinical relevance of findings and associated psychological impact of such screening. Therefore, we conducted a prospective follow-up study to provide insight in the effectiveness and psychological impact of direct-to-consumer screening using both WB-MRI and cardiological examination.

**Methods and findings:**

The study population consisted of 3603 voluntary, primarily middle-aged participants who underwent commercial WB-MRI and cardiological screening at one of 6 study clinics in Germany or the Netherlands between July 2014 and March 2016. MRI investigation consisted of directed scans of the brain, neck, abdomen and pelvis. Cardiovascular examination included pulmonary function, resting electrocardiogram, transthoracic echocardiogram and a bicycle exercise stress test. Findings were assessed by experienced radiologists and cardiologists. In addition, participants were inquired about several (psychological) domains, including the expectations and consequences of the screening procedure. Out of 3603 individuals, 402 (11.2%) demonstrated abnormal MRI (n = 381) and/or cardiological findings (n = 79) for which they were advised to undergo further consultation <3 months in regular healthcare. In 59.1% of cases of abnormal MRI findings which were consulted, fully completed consultations were available in 87.1%. After consultation, 77.6% of initial MRI outcomes were adopted. In 40.9% of cases of abnormal MRI findings, recommendations for consultation were not adhered to during the study period. 71.1% of adopted MRI-findings required treatment or monitoring, including 19 malignancies. For abnormal cardiological findings, 70.9% of cases were consulted in regular healthcare. Of these, 91.1% had a completed follow-up procedure of which 72.5% of initial findings were adopted and 83.8% of these findings required treatment or monitoring. The most frequently reported psychological consequences of the screening procedure were getting reassurance (72.0%) and insight into one’s own health status (83.0%). 5.0% reported to feel insecure about their health and 6.2% worried more about their health as a consequence of screening. Main limitations of the study were considered the telephonic follow-up of referred clients and the heterogeneity of screening equipment and assessment of radiologists and cardiologists.

**Conclusions:**

Direct-to-consumer screening using whole-body MRI and cardiological testing is feasible and effective for the detection of clinically relevant and treatable abnormalities. Psychological harm was not frequently reported in study participants.

## Introduction

Noncommunicable diseases (NCDs) such as cancer and cardiovascular disease are the leading causes of death, accounting for more than 70% of mortality worldwide [1]. Costs of NCDs are responsible for the largest share of healthcare expenditures [2,3], costs for cancer alone are projected to increase to a staggering 158 billion in 2020 which is a 27% increase as compared to 2010 [4,5]. Rising healthcare costs are in part due to the inability of conventional medicine to widely adopt to a more pro-active and preventative organisation.

Current nationwide primary disease-prevention strategies like colorectal, cervical and breast cancer screening are an effective population health tool to reduce cancer mortality and morbidity, although adherence is still far from optimal [5–7]. In contrast, a growing number of individuals chooses to gain insight into their physical wellbeing and utilize the increasing availability of so-called Direct-To-Consumer (DTC) services, like health check-ups, imaging, genomic and/or frequent laboratory testing [8]. DTC services, largely focused on primary prevention, are the commercial offerings of medical products and services directly to individuals by for-profit companies instead of being ordered by conventional healthcare providers.

One DTC service which has gained popularity is the whole-body magnetic resonance imaging (WB-MRI), which is increasingly applied in a clinical and commercial setting [9,10]. However, the application of whole-body imaging as a screening modality has raised considerable debate in both scientific [11,12] and public literature [13] because of unknown benefits and potential harms for the individual and impact to society. In case of no identified abnormalities, this includes risk of false negatives and therefore false reassurances. Identifying abnormal findings conveys a risk of false-positive or clinically irrelevant findings, complications of sometimes burdensome invasive follow-up procedures, as well as (costly) overdiagnosis- and treatment [14–16]. In addition, whole-body imaging may have a psychological impact: causing anxiety in presence of (uncertain) findings or a diminished incentive for lifestyle-improvement in individuals with normal results [15,17].

To provide insight in the effectiveness and psychological effects of commercial direct-to-consumer high-intensity screening, we performed a prospective study in volunteers using WB-MRI combined with an intense cardiovascular work-up.

## Methods

### Study design

The study participants consisted of Dutch individuals utilizing the commercially available direct-to-consumer services (‘total body scans’ or ‘TBS’) offered by a Dutch health services company (Prescan BV). Study participants were prospectively recruited from all consecutive Prescan clients, from all regions in the Netherlands, who were scheduling a TBS between 2014–2015. During the scheduling process, clients were asked if they were interested to participate, after which they were fully informed about the study procedures and the informed consent was signed.

A TBS consists of 4 focused MRIs and cardiovascular screening all targeted at (early) detection of disease and providing insight into an individuals’ health status. The company has developed their services during the past 14 years, and consumers are made aware of the TBS through the following channels: 49.8% through marketing, 32.0% word-of-mouth advertising, 11.0% offered to employees by businesses and 7.3% by other means. All participants were prospectively enrolled between July 2014 and March 2016 while visiting one of 4 study clinics in Germany or 2 in the Netherlands.

The Netherlands Organisation for Applied Scientific Research (TNO) was commissioned by Prescan BV to perform this independent study. TNO is an independent organization regulated by public law which has a focus on improving the well-being of society in a sustainable way (https://www.tno.nl/en/).

### Informed consent

Prior to the screening procedure, all individuals were informed about the benefits, risks and possible consequences of each of the examinations. This included the possibility and implications of false-positive and/or false-negative findings. First, participants gave written informed consent to undergo WB-MRI and cardiological screening, as offered by Prescan BV. Second, separate written informed consent was given for participation in this study. This included that the pseudonymized answers to the questionnaires and interview could be used for research analysis. At the time of the study initiation (2014), these questionnaires did not fall under the remit of the Dutch Act on Research (Medical Research Human Subjects Act; https://english.ccmo.nl/) involving human subjects and the study did not need prior approval by an external ethical review board.

### Study procedures

#### MRI procedure and radiological reading

Each participant underwent an MRI investigation consisting of 4 sequential, directed scans of the brain, neck, abdomen and pelvis. All MRI scans were performed on 1.5T (Rheine, Gronau, Bottrop, Baarn and Schiedam) and 3T (Bocholt) systems. Individuals who agreed to intravenous contrast administration received a gadolinium-based contrast agent (S1 Table). Contrast agents were not administered in case of a poor renal function (eGFR <30 ml/min/1.73m^2^). An overview and detailed description of the scanning procedures used can be found in S2 Table.

MRI imaging data were analysed and interpreted by experienced and certified radiologists specialized in screening and preventive healthcare. Subsequently, participants were provided an examination report and, in case of abnormal findings, a recommendation for additional follow-up examination or treatment within a certain timeframe.

As part of the study procedure, RS classified MRI findings using ICD-10 and SNOMED-CT coding and characterized them as clinically relevant or irrelevant. Potentially clinically relevant findings were considered findings for which referral within 3 months was advised for consultation in the conventional healthcare setting. Relevant findings were thereafter categorized as suspected malignant tumours, tumours with uncertain behaviour (e.g. having characteristics of malignant potential), suspected benign tumours, vascular disease, aneurysms, cystic abnormalities or other. Based on the severity of these clinically relevant findings, the referral group consisted of participants who were referred for consultation within either <1 week (e.g. suspected malignancy) or <3 months (e.g. suspected benign tumour). The non-referral group included participants without a follow-up recommendation, or with advice to repeat the diagnostic procedure after at least more than one year. In addition, participants in the non-referral group who underwent additional examinations (TBS-Plus), e.g.: laboratory testing, CT-scans, gastro- and colonoscopy’s or ultrasounds were excluded from further follow-up. These were examinations not of interest for this study, because of the possibility of additional findings, thus the follow-up of these findings was not included in the digital questionnaires provided to this group. These individuals were excluded from further follow-up. Lastly, clients who received a recommendation for follow-up in between ≥3 months and ≤1 year were excluded from follow-up and analysis in this study. This interval was selected because at time of the study, the clinical relevance of these findings was still uncertain.

#### Cardiovascular examination and assessment

Each participant underwent an extensive cardiovascular examination. First, a standardized interview was conducted focusing on risk factors, lifestyle, general medical history and history and/or symptoms of cardiovascular disease based on a questionnaire developed for preventive cardiological examination. Then, a biometric measurement was done including height, weight, body mass index and a blood pressure measurement. A pulmonary function testing was performed using spirometry and spirograms. Flow volume curves were obtained and FVC, FEV_1_ and FEV_1_/ FVC ratio were calculated. Thereafter, a resting electrocardiogram (ECG) was performed using standard 12-lead placement and a transthoracic echocardiogram (TTE) was conducted using standard transducer orientations. Finally, all participants underwent a bicycle exercise stress test. A more detailed description of cardiovascular equipment used at each centre can be found at S3 Table. All cardiovascular examination data were assessed by experienced and certified cardiologists. As part of this study, abnormalities were thereafter classified using ICD-10 coding and participants were provided with a therapeutic or diagnostic follow-up recommendation.

### Study data

#### Pre-study assessments

All study participants were requested to fill out a written questionnaire at the start of the study period before the screening (pre-measurement). In addition to the intake questionnaire (S1 File), Questions were asked within the following three domains (S1 Fig, S4 Table):

- The expected consequences of the screening procedure were assessed in 2 subdomains: (A1*) insight into your health status* and (A2) *your emotional wellbeing*. Answers were collected using a 3-point scale (yes; maybe; no, or N/A) [18].
- One question about *self-perceived health* was asked on a 5-point scale (ranging from very well to very poor) [19]; and
- Motives for screening were explored using 11 statements provided with 3-point scale (yes; maybe; no, or N/A) for answering, based on the Dutch Preventive Care Guideline [20] and Van Asperen et al [21].

#### Post-study assessments

Seven weeks after the screening procedure, or after (partial) completion of the follow-up process, non-referred clients were digitally, and referred clients telephonically, interviewed about (A1) insight into your health status, (A2) your emotional wellbeing and your (C) self-perceived health. Additionally, in context of (A2) your emotional wellbeing, questions were asked about actual possible negative consequences of the procedure. Furthermore, clients were interviewed about (D) impact on your lifestyle and health status. For referred clients, a telephonic interview was chosen to ensure a thorough and detailed assessment of their follow-up.

#### Follow-up referral group

Referred clients were telephonically interviewed in order to assess follow-up after the screening procedure into detail. Follow-up of each clinically relevant finding was separately explored regarding the type of visited healthcare professional and clinic. Thereafter, questions were asked about outcomes of any additional examinations and consecutive therapeutic actions.

#### Quality assurance and quality control

Two audits were executed by TNO to check the data collected by Prescan. First, a selection of all MRI findings was also coded by an independent assessor (TH). This double coding included assessment of (a) adoption of findings in general healthcare, (b) follow-up diagnostics after referral, and (c) treatment in general healthcare. Second, at least 6 weeks after the initial telephonic interview, audits were performed to verify answers from a random selection of clients in the referral group.

#### Statistical analysis

All statistical analysis were descriptive statistics. Continuous variables were expressed as means with standard deviations, and categorical variables as frequencies and percentage of total. Unpaired t-tests and Chi-square tests were used to assess differences between the referral and non-referral groups, and in case of low expected cell values, Fisher’s exact tests were used. McNemar's or paired samples T-tests were applied for dichotomous and continuous data in assessment of pre- and post-measurement questionnaire data differences. Cohen’s kappa (κ) was assessed as a measure for inter-rater agreement for double coding of the referred MRI findings in the audit. Statistical analysis software used was SPSS (version 23; IBM Corp, Armonk, NY).

“No accurate sample size calculation was feasible since no data on whole-body MRI have been published that were applicable to the TBS. We assumed a cohort size of 500 would be adequate since we were describing a dichotomy proportion in one group, for example, the percentage of clients worried about the TBS outcomes, a width of the 95% confidence interval of 10% is acceptable at an expected proportion of 50%. This means that in a subsequent study the percentage found will be between 45-55%. This includes 400 clients per group.”

## Results

### Study population

**Fig 1:**
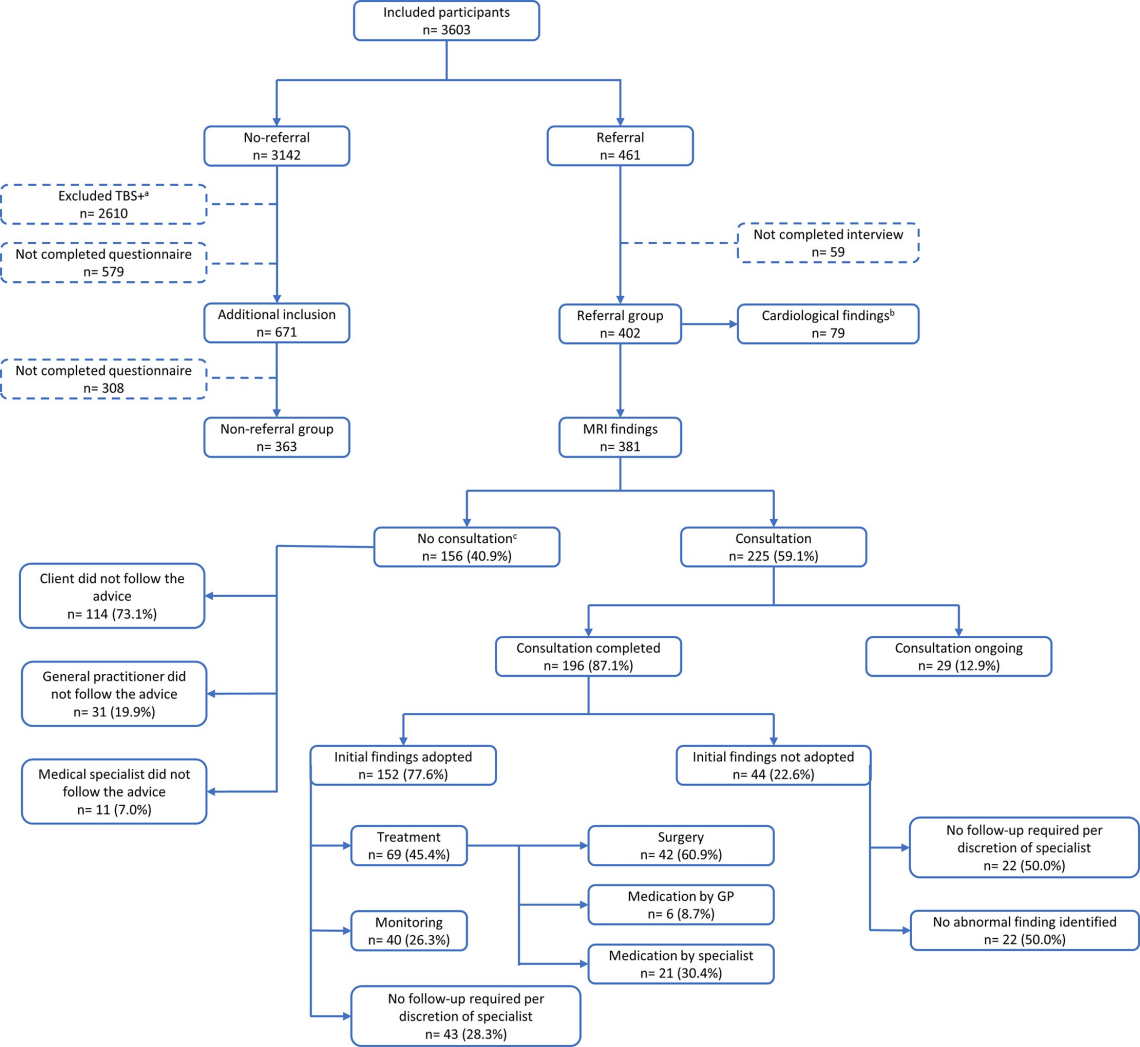
Flowchart of included participants, and overview of follow-up and management of MRI-findings in general healthcare the referral group. ^a^ A TBS+ (Total Body Scan +) is the standard MRI- and cardiovascular screening procedure with optional additional examinations, e.g.: laboratory tests, CT-scans, gastro- and colonoscopy’s or ultrasounds. These clients were excluded (n = 2610) from follow-up in the non-referral group, because of the possibility of (additional) findings with these examinations. ^b^ An overview of follow-up and management of cardiological findings can be found in S2 Fig. ^c^ *‘No consultation’* included clients who did not follow the advice for a consultation <3 months during the study period.

During the study period, 57% of eligible clients agreed to participate. An additional 16% of eligible participants were excluded for a variety of reasons, including a referral between ≥3 months and ≤1 year instead of <3 months. The resulting study population consisted of 3603 participants (Fig 1). After exclusion of individuals because of incomplete questionnaires or follow-up data, the study sample consisted of a total of 765 clients, of which 402 (53%) in the referral and 363 (47%) in the non-referral group. Except for the Bocholt clinic (0 vs. 6.5%), the (non-) referral percentages did not differ between participating clinics (S5 Table). Demographics and lifestyle-related factors are described in Table 1. The mean age of the study sample was 59.6 years (SD 10.7; range 24-84 years), 60% were men and 40% women. This did not differ between referred and non-referred participants. The referred group were in general more often smokers (16.5%) as compared to the non-referral group (8.1%) but smoked less than the general Dutch population. The referred group had a comparable alcohol consumption and BMI, as compared to non-referred group and to the general Dutch population.

**Table 1:** Baseline characteristics: Non-referred versus referred participants, in comparison with the general Dutch population (age >20 years).

	Non-referral (n= 356^a^)	Referral (n= 402)	Dutch Population^b^
**Men, n (%)**	208 (57.3)	240 (59.7)	49.5
**Age, mean (SD), years**	59.1 (10.7)	60.0 (10.7)	41.3
20-40 (%)	4.7	4.5	24.5
40-55	25.3	24.1	22.1
55-70	54.7	53.2	19.0
70≥	14.3	18.2	11.8
**BMI, mean (SD), kg/m^2^**	25.8 (3.5)	26.2 (3.9)	25.7
<18,5 (%)	0.8	1.0	1.6
18,5-24,9	43.3	41.8	48.1
25-29,9	45.6	42.8	36.6
30>	10.2	14.4	13.7
**Lifestyle**			
Alcohol consumers (%)	76.7	80.5	80.4
Current smokers (%)	8.1	16.5	26.0
History of smoking (%)	20.4	24.4	N.A.

Abbreviations: SD, standard deviation; BMI, body mass index; N.A., not applicable.

^a^ Incomplete baseline characteristics data for 7 non-referred participants, thus characteristics are based on 356 in the non-referral group.

b Dutch population characteristics are based on data from 2015 the Dutch national statistical office (CBS; available at https://statline.cbs.nl)

### MRI outcomes

**Fig 2:**
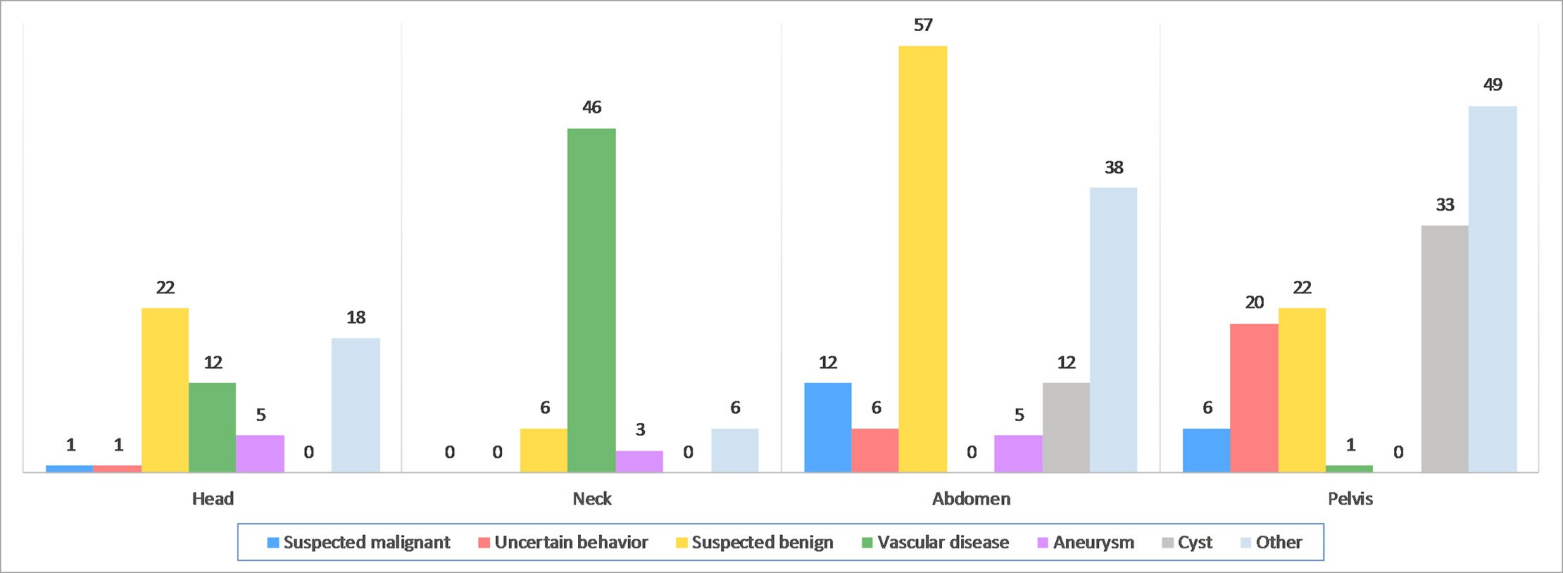
Categorization of MRI findings (n = 381) in the referral group subdivided by type of MRI investigation. At radiological evaluation, neoplasms were considered either suspected malignant tumors, tumors with uncertain behavior (i.e. having characteristics of malignant potential) or suspected benign tumors. All observed vascular disease in the neck region (n = 46) were occlusion and stenosis of the carotid arteries (n = 38, 30-70% stenosis; n=6, >70% stenosis). A large proportion of ‘other’ findings were for example gallbladder abnormalities (n = 7) and hepatic hemangioma’s (n = 13) in the abdomen, and benign prostatic hyperplasia (n =34) in the pelvis.

Fig 1 depicts the outcomes of the MRI findings in the referral group. Out of the 3603, a total of 461 (12.8%) participants had abnormal findings in either the MRI or cardiovascular screening procedure. After exclusion of participants due to incomplete questionnaires or follow-up data, the total referral group consisted of 402 participants. Of those, 460 abnormal findings were considered clinically relevant: 381 on MRI and 79 during cardiological testing. A total of 305 participants had one and 36 had at least two abnormal findings on MRI (S6 Table). A total of 153 tumours (40.1%) were identified. These included 19 (12.4%) suspected malignancies, 27 (17.6%) tumours with uncertain behaviour and 107 (69.9%) suspected benign tumours. The majority of tumours were localised in the abdomen or pelvis (Fig 2), of which the most common findings were suspected benign kidney tumours (n = 22) and neoplasms of uncertain behaviour of the prostate (n = 14) (S7 Table).

In 40.9% (156) of abnormal findings, recommendations for consultation in conventional healthcare were not adhered to. Decisions for non-adherence were made in 73.1% by participants themselves, in 19.9% by general practitioners’ and in 7.0% by medical specialists. These findings were re-analysed by an experienced radiologist (GK) to assess whether these decisions to not adhere to the recommendations were appropriate. In 121 (80.7%) of the findings it appeared that based upon MRI evaluation, participants were initially correctly referred for consultation in conventional healthcare. Afterwards, 68.5% of these clients turned out to eventually have visited their general practitioner as a consequence of screening.

**Fig 3:**
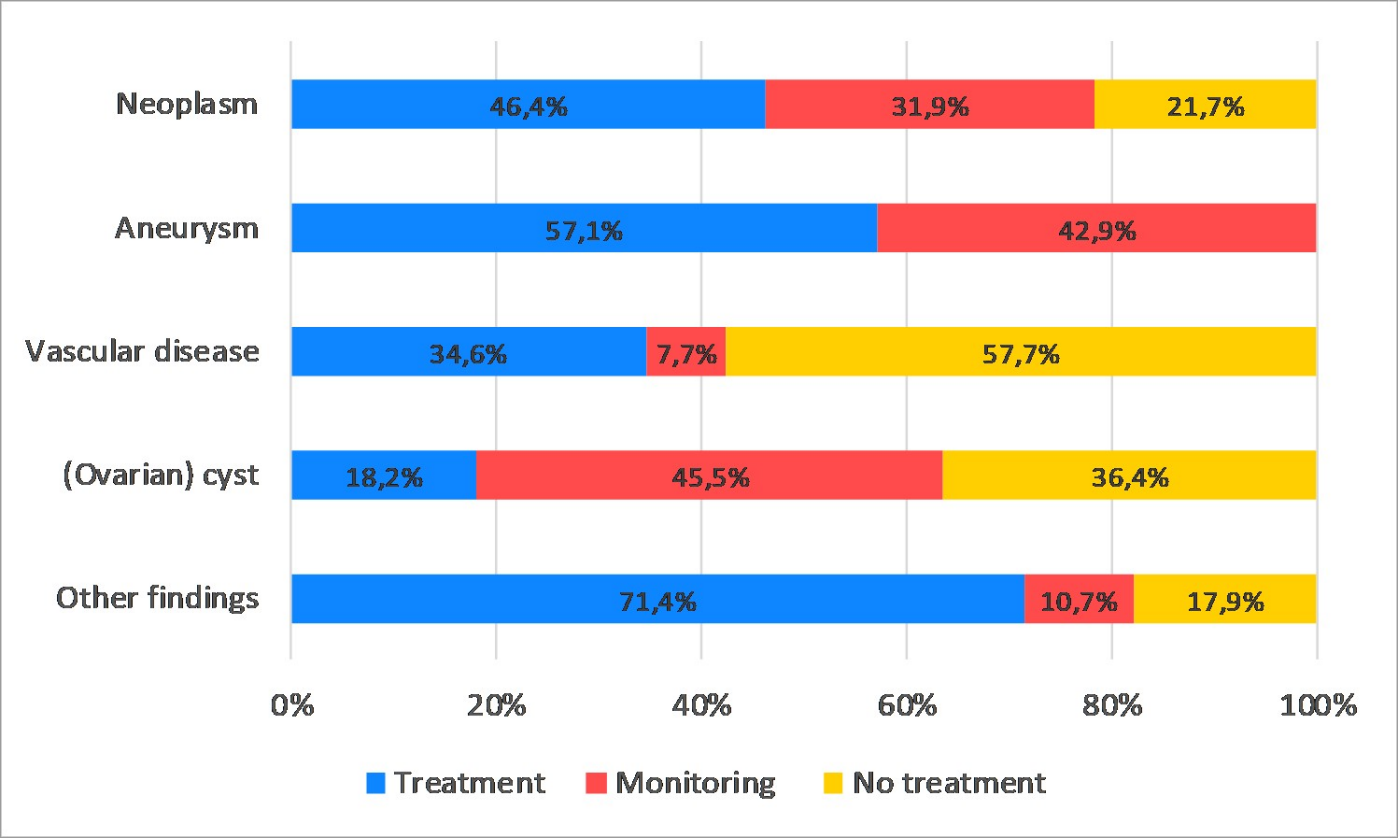
Overview of management (treatment, monitoring or no treatment) of MRI findings in the referral group (n = 381), subdivided by type of finding. After confirmation in general healthcare, neoplasms consisted of malignant tumors (n = 19), benign brain tumors (n = 15) or benign tumors localized elsewhere in the body (n = 35). A total of 7 aneurysms, 26 vascular disease and 22 (ovarian) cysts were identified. Other findings (n = 28) consisted of e.g. chronic sinusitis (n = 4), pneumonia (n = 3), and ureteral stone with hydronephrosis (n = 3).

In the remaining 225 (59.1%) of abnormal findings, consultation was completed in 196 (87.1%) cases. Consultation frequently consisted of repeat imaging to verify the MRI abnormality: ultrasound in 32.0%, MRI in 12.4% and a CT-scan in 12.0%. Biopsies and endoscopies were performed in 9.3% and in 4.9% of cases, respectively. Overall, 152 (77.6%) of all initial MRI recommendations were adopted and in 44 (22.6%) the initial findings were not adopted. In 22 out of 44 findings, the initial abnormality was not observed in follow-up imaging. In 45.4% of cases, where the initial recommendations were adopted, treatment was initiated (Figs 1 and 3, S8 Table). In the remaining 26.3% of cases, clinical monitoring was judged necessary since future treatment was considered likely (e.g. aneurysm; small brain tumour). Of the 153 neoplasms discovered by WB-MRI, 69 findings (45.1%) were adopted, constituting 19 malignant tumours, 15 benign brain tumours and 35 benign tumours localized elsewhere in the body.

### Cardiovascular outcomes

**Fig 4:**
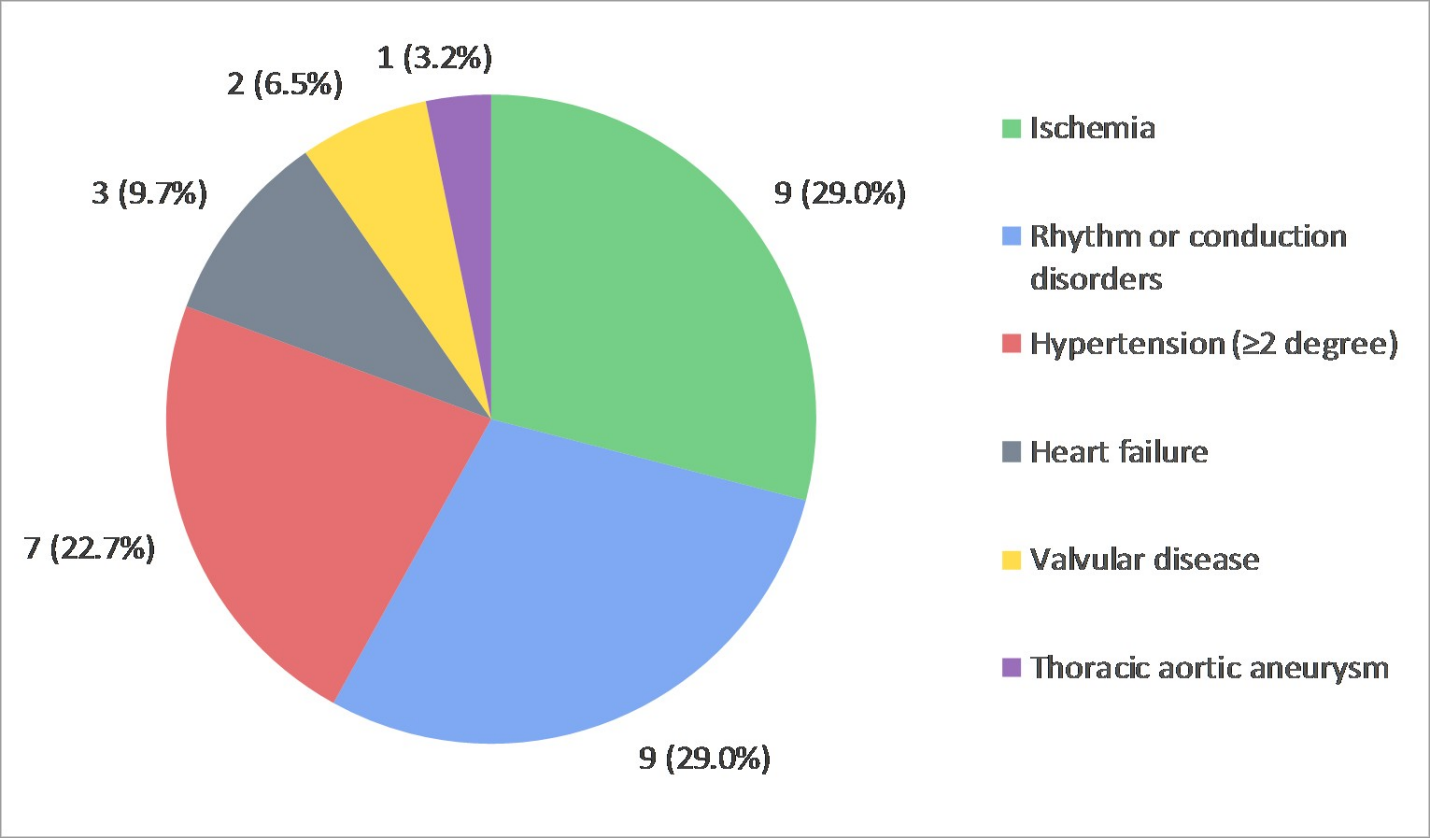
Overview of all cardiovascular findings (n =31) requiring treatment or monitoring after adoption in general healthcare. A total of 79 findings were primarily identified, in which in 70.9% of the cases, follow-up recommendations for consultation were followed. In conclusion, a total of 37 (72.5%) of the initial findings were adopted in follow-up (S2 Fig).

Following cardiological screening, a total of 79 abnormal findings were referred, which were mainly identified during ECG (n = 24, 30.4%), exercise stress testing (n = 22, 27.8%) and TTE (n = 15; 19.0%) examination (S2 Fig and S9 Table). In 70.9% (56) of the findings, recommendations for follow-up consultation were adhered to and in the majority of the findings (91.1%) completed during the study period. Subsequently, 72.5% (37) of the findings were adopted in conventional healthcare. Fig 4 shows that frequently adopted findings requiring treatment or monitoring (n = 31, 83.8%) in general healthcare were signs or symptoms of myocardial ischemia (n = 9, 29.0%), rhythm or conduction disorders (n = 9, 29.0%) and hypertension (n = 7, 22.7%).

### Psychological impact

**Fig 5:**
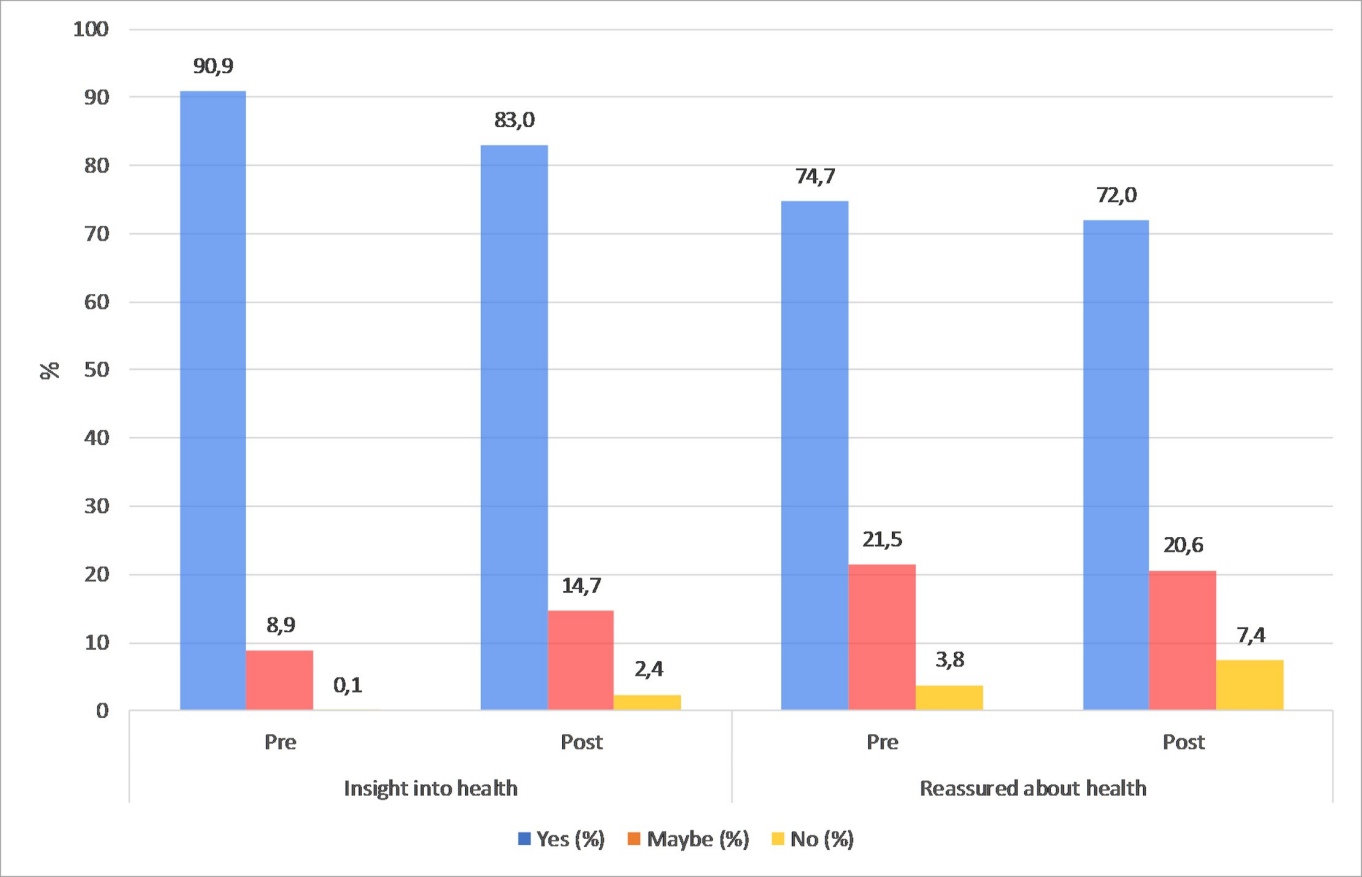
**(A) *Expected* (pre) and *actual* (post) consequences regarding (A1) insight into health status and (A2) being reassured about health (Fig 1).** Depicted in the figure are the percentage of participants who answered “yes, maybe or no” for both the pre- and post-measurement (N/A not included).

The following items were addressed by both referred and non-referred clients, excluding the ‘not-applicable’ answers:

- Expected and actual consequences of the screening procedure: 90.9% of the participants expected to gain insight into their health status and 74.7% expected to get reassured about their health status. After screening, 83.0% and 72.0%, respectively, indicated they actually experienced these consequences (Fig 5). Regarding possible negative emotional consequences of the screening procedure (A2), very few participants (0.7%, 5/708) regretted their participation. Furthermore, 5.0% (35/702) reported to feel insecure about their health and 6.2% (41/665) worried more about their health.
- Pre- and post-screening self-perceived health: prior to the screening procedure, 73.0% of the participants reported their self-perceived health status to be “(very) good”. This is less than reported in the Dutch population (79.5%) and was more distinct in referred clients (69.2%) as compared to non-referred clients (77.3%). After the screening procedure, a statistically significant increase was observed for referred (69.2% vs. 82.0%, p < 0.001), but not for non-referred clients (vs. 77.3% vs. 81.0%, p = 0.092).
- Motives for screening: most important motives for screening were “wanting clarity about my health status” (96.4%, 691/717) and “in case of disease, I want to be aware (as early as possible)” (95.5%, 693/726). In addition, 65.7% (283/431) of participants reported that physical complaints were a reason for screening.
- Impact on lifestyle and health status: after the screening procedure, the following lifestyle changes were reported: a 22% decrease of smoking, 17% increase of healthier diet, 16% more physical activity, 12% weight reduction and 8% decrease of alcohol consumption.

### Quality assurance and quality control

The quality control for the coding of the findings in the referral group showed satisfactory results. For the 69 double coded MRI findings, good inter-rater agreement was observed for coding of (a) confirmation of findings in general healthcare (κ = 0.67), (b) follow-up diagnostics after referral (κ = 0.73), and (c) subsequent treatment in general healthcare (κ = 0.77).

For the quality control of the interviews with referred clients, 39 clients were approached of which 23 participated in the audits, corresponding with 5.7% of the referred clients in total. In the audits, minor differences were observed in the answers provided by clients in the telephonic interviews and therefore TNO concluded that the obtained data were reliable.

## Discussion

In this large and unique prospective study of clients in a commercial direct-to-consumer high-intensity screening setting, we provided an overview of the frequency, type and management of abnormal MRI and/or cardiological findings accompanied with an exploration of the psychological impact and consequences of this type of screening.

In 12.8% of a total of 3603 mostly middle-aged individuals, abnormal findings were identified and referred for consultation in conventional healthcare. For abnormal MRI findings, more than three quarters were adopted, of which the majority required treatment or follow-up. Neoplasms constituted 45% (69/152) of adopted findings, among which 19 malignancies and 15 brain tumours for which treatment or monitoring was required. For cardiological screening, the most frequent findings were ischemia, rhythm or conduction disorders and hypertension, requiring treatment or monitoring in more than 80% of the cases in which the diagnosis was confirmed. An important observation is that in a large proportion of findings (40.9% of MRI findings and 29.1% of cardiological findings) recommendations for consultation in general healthcare were not adhered to during the study period. Studies to explain this observation are currently ongoing.

Regarding psychological aspects, the majority of participants expected to gain insight into their health status or reassurance about their health status which they indeed confirmed following the procedures and digesting the outcomes. This study did not observe a significant psychological impact of this type of screening. Negative emotions like feelings of insecurity or being more worried about their health as a consequence of screening were infrequent, 5.0% and 6.2% respectively. Studies to investigate the long-term psychological impact are planned.

Previous studies on WB-MRI focused primarily on technical feasibility and prevalence of abnormalities in selected populations [22–24], or examined the presence of incidental findings (IFs) in a clinical- or research setting [11,25–27]. IFs, sometimes called incidentaloma’s, can be defined as *findings that are discovered by chance in the context of radiological diagnostics which can potentially affect the health of an individual* [28]. Different from finding incidentaloma’s, this study was purposely designed to identify these (asymptomatic) abnormalities, allowing intervention in an early stage and possibly saving lives. Comparing our results to a recent meta-analysis on prevalence and severity of IFs from Gibson et al. [10], we have found a similar prevalence of potentially serious and indeterminate findings combined (pooled prevalence: 12.8% versus 10.6%). However, our results might be best comparable to Hegenscheid et al. [26], who investigated IFs in a general population cohort consisting of middle-aged individuals. They reported a higher rate of potentially relevant findings (32%) and possible malignancies (5.9%). However, their protocol was designed from a research perspective and was not optimized for providing clinical information. In addition, no follow-up data were available.

Part of the debate on the benefits versus harms of whole-body screening is the possibility of triggering negative psychosocial consequences, such as anxiety and distress. Our results do not support this, as overall approximately 5.5% reported to feel insecure or worried more about their health as a consequence of screening, and only 0.7% regretted their participation. Meanwhile, a growing number of individuals chooses to take initiative in their physical and mental wellbeing and seek insight into their health status and reassurance about their complaints [12,29,30]. This is supported by our findings, where insight into health and reassurance about their health were important expectations and consequences of screening. In our observations these expectations were in almost all cases fulfilled, but less often than anticipated beforehand. As reported by Schmidt et al. [12], this may be a consequence of an overestimation of the personal benefits of screening, like expecting to gain a complete overview of their physical condition.

The strengths of our study include the unique insight in a commercial screening enterprise with a large sample of health consumers. The prospective nature of our study made it possible to collect detailed information on types, frequencies, follow-up of findings, and in addition provide insight in the psychological impact of intense screening. This may be valuable information to communicate to prospective clients to make more informed decisions [31,32]. Furthermore, these data will likely contribute in the ongoing debate about harms versus benefits of screening.

Our study has several limitations. First, the participating study centres were heterogeneous with regards to MRI and cardiological equipment, MRI sequences, use of gadolinium-contrast, and the likely variation of participating radiologists and cardiologists. As a consequence, this may have resulted in varying conclusions about the nature and need for referral. However, 1.5 Tesla and 3.0 Tesla MRI systems seem to be comparable in terms of diagnostic quality [33,34] and referral rates did not differ between centres. Therefore, albeit speculation at this time, it may have also resulted in better generalizability for future clinical practice. Second, our design may be prone to several sources of bias. A proportion of non-referred clients did not complete their follow-up questionnaires. This may have led to over-representation of a selection of non-referred clients, who were possibly more satisfied with (the outcomes of) the screening procedure. Regarding referred clients, the self-reported nature of their follow-up which required a telephonic interview made it vulnerable to both recall and interpretation bias. Unfortunately, we did not have the possibility to acquire medical reports, therefore we feel that a telephonic interview was the most suitable method to obtain a thorough and detailed overview of the (sometimes complex) follow-up of their findings. Third, in retrospect, we feel that it would have been appropriate to construct an à priori classification of findings which would have been considered clinically relevant versus irrelevant, providing a guideline for referring radiologists and cardiologists. Gibson et al. [10] provide an excellent example of such, used for classifying IFs as potentially serious, or non-serious. Finally, this study was not designed to study the health economic impact of this type of screening which is often thought to consume unnecessary healthcare resources during follow-up examinations.

In conclusion, we found that high intensity WB-MRI and cardiological screening in a direct-to-consumer setting leads to the identification of clinically relevant and treatable abnormalities in asymptomatic individuals without causing psychological distress.

## Supporting information

S1 Checklist (DOC)

S1 Fig Pre- and post-measurement questionnaire domains. (DOCX)

S2 Fig Follow-up of cardiological findings in the referral group. (DOCX)

S1 Table MRI contrast agents used per participating center. (DOCX)

S2 Table MRI machine characteristics. (DOCX)

S3 Table Cardiovascular examination equipment overview. (DOCX)

S4 Table Pre- and post-measurement questionnaires. (DOCX)

S5 Table Percentages of non-referred and referred clients, and total number of clients included per study clinic. (DOCX)

S6 Table Number of findings in MRI and cardiological examination per participant. (DOCX)

S7 Table Categorization of MRI findings (n = 381) in the referral group subdivided by localization. (DOCX)

S8 Table Type of confirmed MRI finding requiring treatment or monitoring. (DOCX)

S9 Table Number of cardiological findings (n = 79) per type of diagnostic examination. (DOCX)

S1 File Intake questionnaire. (DOCX)

S2 File (PDF)
