# Supplementary material for: Health outcomes and experiences of direct-to-consumer high-intensity screening using both whole-body magnetic resonance imaging and cardiological examination
Source: PLoS One. 2020 Nov 20;15(11):e0242066. doi: 10.1371/journal.pone.0242066 (PMC7678982; doi:10.1371/journal.pone.0242066)

**S1 Fig.** Overview of pre- and post-measurement questionnaire domains. (A) *Expected* and *actual* impact and consequences consisting of 2 subdomains: (A1) insight into health status and (A2) emotional wellbeing. Other questionnaire domains were pre- and post-screening (B) self-perceived health, (C) motives for screening (pre), and (D) impact on lifestyle and health status (post).
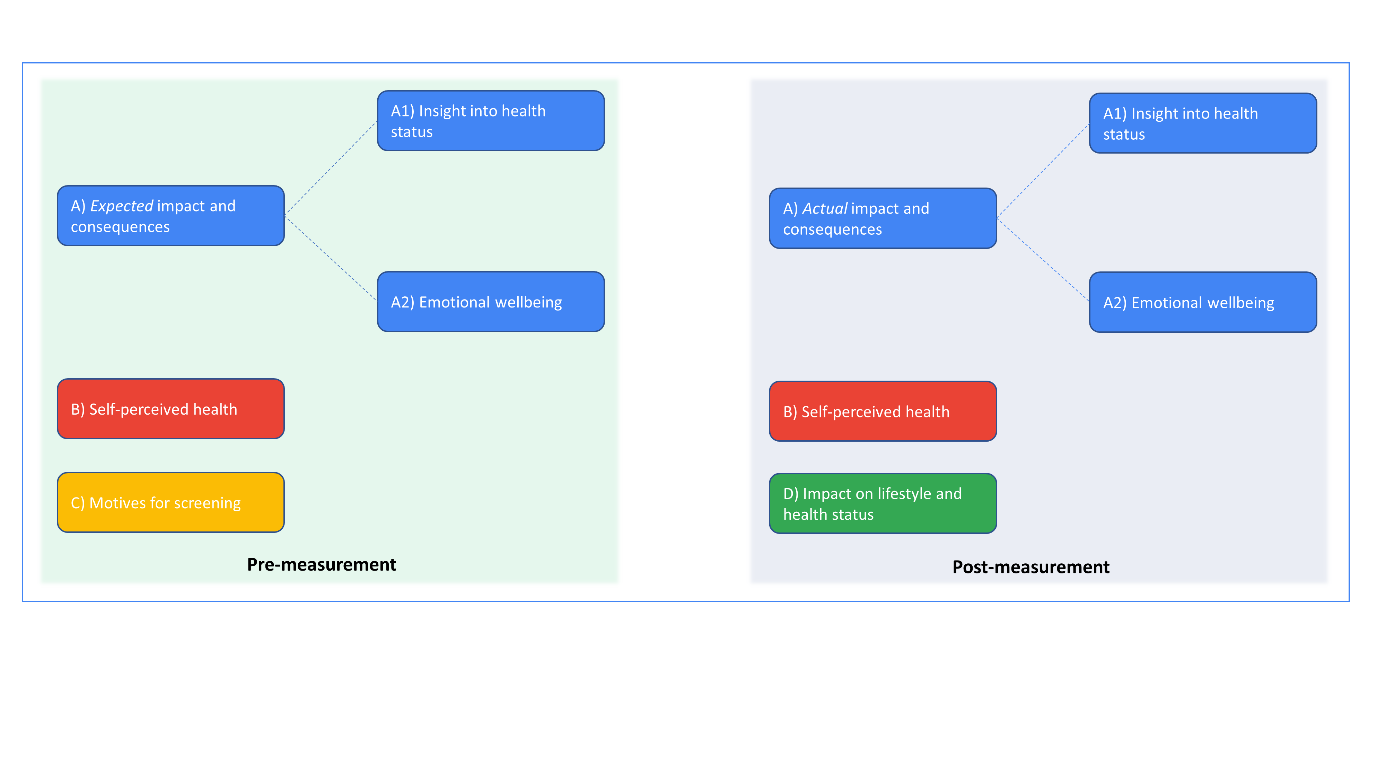

Supplement: S1 Fig — (DOCX) [file pone.0242066.s002.docx]
