# Supplementary material for: Health outcomes and experiences of direct-to-consumer high-intensity screening using both whole-body magnetic resonance imaging and cardiological examination
Source: PLoS One. 2020 Nov 20;15(11):e0242066. doi: 10.1371/journal.pone.0242066 (PMC7678982; doi:10.1371/journal.pone.0242066)

**S2 Fig.** Overview of follow-up and management of cardiological findings in general healthcare in the referral group.


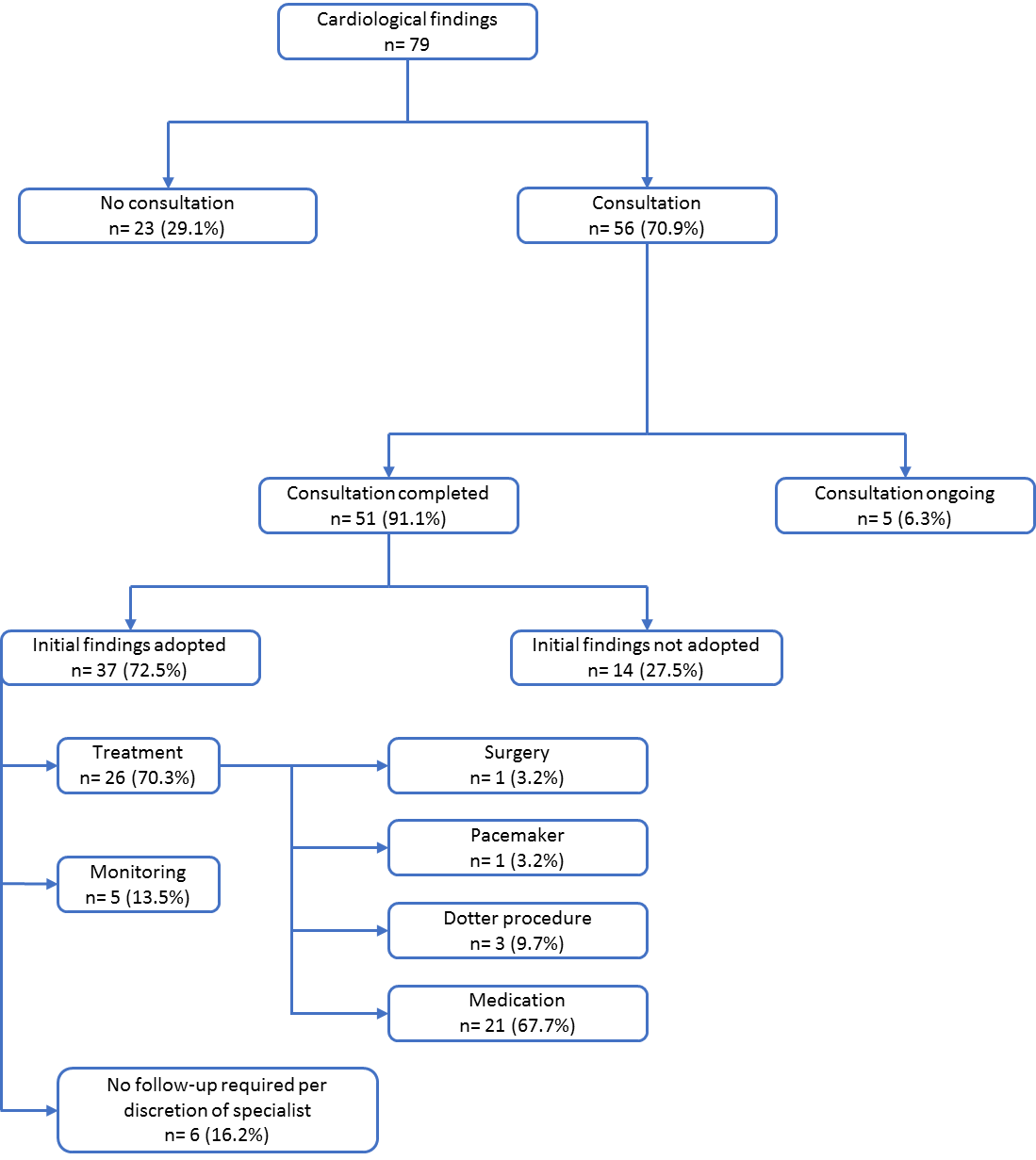

Supplement: S2 Fig — (DOCX) [file pone.0242066.s003.docx]
