# Supplementary material for: Health outcomes and experiences of direct-to-consumer high-intensity screening using both whole-body magnetic resonance imaging and cardiological examination
Source: PLoS One. 2020 Nov 20;15(11):e0242066. doi: 10.1371/journal.pone.0242066 (PMC7678982; doi:10.1371/journal.pone.0242066)
Supplement: S1 Table — (DOCX) [file pone.0242066.s004.docx]

**S1 Table.** Type of MRI contrast agents used per participating center.

| **Center** | **Contrast agent** | **Concentration (mg/mL)** | **Brand name** | **Producer** |
| --- | --- | --- | --- | --- |
| Rheine | Gadoteridol | 279,32 | Prohance® | Bracco |
| Gronau | Gadoterate | 279,32 | Dotarem® | Guerbet |
| Bottrop | Gadoterate | 279,32 | Dotarem® | Guerbet |
| Bocholt* | N.A. | - | - | - |
| Baarn | N.A. | - | - | - |
| Schiedam | N.A. | - | - | - |

* Data on used equipment is not available anymore because the collaboration has stopped.
