# Supplementary material for: Health outcomes and experiences of direct-to-consumer high-intensity screening using both whole-body magnetic resonance imaging and cardiological examination
Source: PLoS One. 2020 Nov 20;15(11):e0242066. doi: 10.1371/journal.pone.0242066 (PMC7678982; doi:10.1371/journal.pone.0242066)
Supplement: S2 Table — (DOCX) [file pone.0242066.s005.docx]

**S2 Table.** MRI brand and type, use of contrast, magnet strength (Tesla) and sequences used per body part for each of the participating centers. Columns marked in grey display availability of contrast and sequences.

|  |  | |  |  | **Head** | | | | | **Neck** | | | | **Abdomen** | | | | **Pelvis** | | |
| --- | --- | --- | --- | --- | --- | --- | --- | --- | --- | --- | --- | --- | --- | --- | --- | --- | --- | --- | --- | --- |
| **Center** | **Brand and type** | **Tesla** | | **Contrast** | **T1** | **T2** | **TSE** | **DWI** | **FLAIR** | **T1** | **T2** | **GE** | **TSE** | **T1** | **T2** | **GE** | **TSE** | **T1** | **T2** | **TSE** |
| Rheine | Siemens Symphony | 1.5 | |  |  |  |  |  |  |  |  |  |  |  |  |  |  |  |  |  |
| Gronau | Philips Achieva | 1.5 | |  |  |  |  |  |  |  |  |  |  |  |  |  |  |  |  |  |
| Bottrop^†^ | 1. GE Signa | 1.5 | |  |  |  |  |  |  |  |  |  |  |  |  |  |  |  |  |  |
|  | 2. Philips Achieva | 1.5 | |  |  |  |  |  |  |  |  |  |  |  |  |  |  |  |  |  |
| Bocholt | Philips Ingenia | 3.0 | |  |  |  |  |  |  |  |  |  |  |  |  |  |  |  |  |  |
| Baarn | Philips Intera | 1.5 | |  |  |  |  |  |  |  |  |  |  |  |  |  |  |  |  |  |
| Schiedam | Siemens Essenza | 1.5 | |  |  |  |  |  |  |  |  |  |  |  |  |  |  |  |  |  |

**^†^** In Bottrop, two different types of MRI scanners were used: 1. GE Signa 1.5 Tesla and 2. Philips Achieva 1.5 Tesla.

**Abbreviations:** TSE = Turbo Spin Echo, DWI = Diffusion Weighted Imaging, FLAIR = Fluid Attenuated Inversion Recovery, GE = Gradient Echo.
