# Supplementary material for: Health outcomes and experiences of direct-to-consumer high-intensity screening using both whole-body magnetic resonance imaging and cardiological examination
Source: PLoS One. 2020 Nov 20;15(11):e0242066. doi: 10.1371/journal.pone.0242066 (PMC7678982; doi:10.1371/journal.pone.0242066)
Supplement: S3 Table — (DOCX) [file pone.0242066.s006.docx]

**S3 Table.** Cardiovascular examination equipment used in each of the participating centers.

| **Center** | **Electrocardiogram** | **Exerice stress test** | **Echocardiogram** | **Spirometry** |
| --- | --- | --- | --- | --- |
| Rheine | Welch Allyn DT100 | Masterscreen CPX | Philips iE33 | CareFusion |
| Gronau | Welch Allyn DT100 | Welch Allyn | Siemens Acuson X600 | Welch Allyn Cardioperfect |
| Bottrop | GE Medical | GE Healthcare | Siemens Acuson CV70 | GE Healthcare |
| Bocholt* | N.A. | - | - | - |
| Baarn | Welch Allyn DT100 | Welch Allyn | Siemens Acuson X300 | Welch Allyn Cardioperfect |
| Schiedam | Welch Allyn DT100 | Welch Allyn | Siemens Acuson X300 | Welch Allyn Cardioperfect |

* Data on used equipment is not available anymore because the collaboration has stopped.
