# Supplementary material for: Health outcomes and experiences of direct-to-consumer high-intensity screening using both whole-body magnetic resonance imaging and cardiological examination
Source: PLoS One. 2020 Nov 20;15(11):e0242066. doi: 10.1371/journal.pone.0242066 (PMC7678982; doi:10.1371/journal.pone.0242066)
Supplement: S4 Table — (DOCX) [file pone.0242066.s007.docx]

**S4 Table.** Overview of the questions asked pre- and post-measurement in each of the questionnaire domains. The colours correspond with the questionnaire domain colours as depicted in Figure 1. For domains A, C and D, answers could be given on a 3-point scale: yes – maybe – no; for domain B, answers could be given on a 5-point scale: very well – good – fair – poor – very poor. Questions in domain A were adopted from Van Dijk et al.^15^, and domain C from the Dutch Preventive Care Guideline^16^ and Van Asperen et al.^17^.

| **Pre-measurement** | **Post-measurement** |
| --- | --- |
| **A) Impact and consequences** | **A) Impact and consequences** |
| *As a consequence of the screening procedure, I expect to…* | *As a consequence of the screening procedure, I…* |
| **A1) Insight into health status** | **A1) Insight into health status** |
| Get insight into my health status | Have gained insight into my health status |
| Get clarity about my complaints | Have gained clarity about my complaints |
| **A2) Emotional wellbeing** | **A2) Emotional wellbeing** |
| Be less worried about my health | Am less worried about my health |
| Be reassured about my health | Am reassured about my health |
|  | Am more worried about my health |
|  | Regret that I did the screening procedure |
|  | Feel insecure about my health |
|  | Feel relieved about my health |
| **A3) Health related behavior** | **A3) Health related behavior** |
| Have a focused approach to my complaints | Have a focused approach to my complaints |
| Know if I can continue (some of) my unhealthy habits | Know that I can continue (some of) my unhealthy habits |
| **B) Self-perceived health** | **B) Self-perceived health** |
| How would you rate your general health status? | How would you rate your general health status? |
| **C) Motives for screening** | **D) Impact on lifestyle and health status** |
| Wanting clarity about my health status | *As a consequence of the screening procedure, I…* |
| I think I am healthy and I want to know this for sure | Have started exercising more frequently |
| In the case of disease, I want to be aware (as early as possible) | Have reduced smoking |
|  | Have reduced my alcohol intake |
| My complaints were not solved in general care | Have started eating healthier |
| I have a family history of certain diseases, and I want to exclude these | Have lost weight |
| With these physical complaints I cannot go to general healthcare (anymore) | *As a consequence of the screening procedure…* |
| I want to know the cause of my complaints | My health status has improved |
| I have an increased risk of cardiovascular disease, I want to know whether this has resulted in any damage | I will live longer |
| I want to know if my unhealthy lifestyle has resulted in any damage | My complaints which were a reason for screening, have reduced |
| I can go to this type of screening quicker, than to general care | My complaints which were a reason for screening, will reduce as a result of the initiated treatment |
| I am afraid that I will regret it afterwards, if I do not do it |  |
