# Supplementary material for: Health outcomes and experiences of direct-to-consumer high-intensity screening using both whole-body magnetic resonance imaging and cardiological examination
Source: PLoS One. 2020 Nov 20;15(11):e0242066. doi: 10.1371/journal.pone.0242066 (PMC7678982; doi:10.1371/journal.pone.0242066)
Supplement: S5 Table — (DOCX) [file pone.0242066.s008.docx]

**S5 Table.** Percentages of non-referred and referred clients, and total number of clients included per study clinic (DE: Germany, NL: Netherlands).

|  | **Non-referral  n= 363 (%)** | **Referral**  **n= 402 (%)** | **Total number of clients** |
| --- | --- | --- | --- |
| Rheine (DE) | 38.9 | 31.6 | 265 |
| Baarn (NL) | 33.2 | 28.9 | 234 |
| Bottrop (DE) | 16.6 | 21.1 | 144 |
| Schiedam (NL) | 6.2 | 6.2 | 47 |
| Gronau (DE) | 5.1 | 5.7 | 41 |
| Bocholt (DE) | 0 | 6.5 | 26 |
|  |  |  |  |
