# Supplementary material for: Health outcomes and experiences of direct-to-consumer high-intensity screening using both whole-body magnetic resonance imaging and cardiological examination
Source: PLoS One. 2020 Nov 20;15(11):e0242066. doi: 10.1371/journal.pone.0242066 (PMC7678982; doi:10.1371/journal.pone.0242066)
Supplement: S6 Table — (DOCX) [file pone.0242066.s009.docx]

**S6 Table.** Number of findings in MRI and cardiological examination per client

|  | Cardiology findings | | | |  |
| --- | --- | --- | --- | --- | --- |
| MRI | 0 | 1 | 2 | 3 | *Total* |
| 0 | - | 58 | 3 | 0 | 61 |
| 1 | 295 | 8 | 2 | 0 | 305 |
| 2 | 30 | 2 | 0 | 0 | 32 |
| 3 | 3 | 1 | 0 | 0 | 4 |
| *Total* | 323 | 69 | 5 | 0 | 402 |
