# Supplementary material for: Health outcomes and experiences of direct-to-consumer high-intensity screening using both whole-body magnetic resonance imaging and cardiological examination
Source: PLoS One. 2020 Nov 20;15(11):e0242066. doi: 10.1371/journal.pone.0242066 (PMC7678982; doi:10.1371/journal.pone.0242066)
Supplement: S7 Table — (DOCX) [file pone.0242066.s010.docx]

**S7 Table.** Categorization of MRI findings (n= 381) in the referral group subdivided by localization.

|  | Head | Neck | Abdomen | Pelvis | Total, n (%) |
| --- | --- | --- | --- | --- | --- |
| Tumour, suspected malignant  Tumour, uncertain behaviour  Tumour, suspected benign  Cyst  Signs of vascular disease  Aneurysm  Other | 1  1  22  0  12  5  18 | 0  0  6  0  46  3  6 | 12  6  57  12  0  5  38 | 6  20  22  33  1  0  49 | 19 (4.9)  27 (7.1)  107 (28.0)  45 (12.0)  59 (15.5)  13 (3.4)  111 (29.1) |
| Total, n (%) | 59 (15.5) | 61 (16.0) | 130 (34.1) | 131 (34.4) | 381 |
