# Supplementary material for: Health outcomes and experiences of direct-to-consumer high-intensity screening using both whole-body magnetic resonance imaging and cardiological examination
Source: PLoS One. 2020 Nov 20;15(11):e0242066. doi: 10.1371/journal.pone.0242066 (PMC7678982; doi:10.1371/journal.pone.0242066)
Supplement: S8 Table — (DOCX) [file pone.0242066.s011.docx]

**S8 Table.** Type of confirmed MRI finding requiring treatment or monitoring.

| Treatment | | Monitoring | |
| --- | --- | --- | --- |
| Type | n (%) | Type | n (%) |
| Tumor | 32 (46.4) | Tumor | 22 (55.0) |
| Malignancy | 19 (27.5) | Aneurysm | 3 (7.5) |
| Aneurysm | 4 (5.8) | Vascular disease | 2 (5.0) |
| Vascular disease | 9 (13.0) | Cyst | 10 (25) |
| Ovarium cyst | 4 (5.8) |  |  |
| Sinusitis | 4 (5.8) |  |  |
| Prostate other | 6 (8.7) |  |  |
| Other | 10 (14.5) |  |  |
| Total | 69 |  | 40 |
