# Supplementary material for: Health outcomes and experiences of direct-to-consumer high-intensity screening using both whole-body magnetic resonance imaging and cardiological examination
Source: PLoS One. 2020 Nov 20;15(11):e0242066. doi: 10.1371/journal.pone.0242066 (PMC7678982; doi:10.1371/journal.pone.0242066)
Supplement: S9 Table — (DOCX) [file pone.0242066.s012.docx]

**S9 Table.** Number of cardiological findings (n= 79) per type of diagnostic examination.

| **Diagnostic examination** | **Number** |
| --- | --- |
| Interview | 3 |
| Blood pressure measurement | 13 |
| Electrocardiogram (ECG) | 24 |
| T.. T.. Echocardiography (TTE) | 15 |
| Stress test | 22 |
| Pulmonary function | 2 |
| *Total* | 79 |
|  |  |
