# Supplementary material for: Health outcomes and experiences of direct-to-consumer high-intensity screening using both whole-body magnetic resonance imaging and cardiological examination
Source: PLoS One. 2020 Nov 20;15(11):e0242066. doi: 10.1371/journal.pone.0242066 (PMC7678982; doi:10.1371/journal.pone.0242066)
Supplement: S1 File — (DOCX) [file pone.0242066.s013.docx]

**S1 File.** Intake questionnaire.

1. Age
2. Length
3. Weight
4. Work (occupation)
5. Are you a smoker or have you smoked? If so, specify what, how much and for how long? If stopped, specify how much smoked, how long stopped and since when.
6. Alcohol consumption? If Yes, how much?
7. Do you have diabetes? If so svp. mention what type (think of medication such as insulin/tuning doctor)
8. Indicate which diseases are known in the family and at what age the condition occurred and also indicate whether it is first degree (children, parents, siblings) or second-degree (uncles, aunts, their children and grandparents) family.
9. Have you been to Prescan before? If so, were there any relevant findings made at the time?
10. Have you undergone tests in the last two years? If so, what investigations?
11. Are there any physical complaints at the moment? (e.: chest pain, fatigue, shortness of breath, abdominal pain, joint complaints or is there a joint replacement?)
12. Have you had surgery in the past? (if yes, please specify)
13. Are you familiar with a (drug) allergies?
14. Are you taking medications such as blood thinners, heart medication, cholesterol reductions, NSAID (ibuprofen/diclofenac) or other medications? S.v.p. indicate which medications and dosage.
15. Have you experienced any illnesses in the past? Or are you familiar with a chronic disease?
16. Known with elevated cholesterol levels - for which medication?
17. Known with elevated blood pressure - for which medication?
