# Supplementary material for: Health outcomes and experiences of direct-to-consumer high-intensity screening using both whole-body magnetic resonance imaging and cardiological examination
Source: PLoS One. 2020 Nov 20;15(11):e0242066. doi: 10.1371/journal.pone.0242066 (PMC7678982; doi:10.1371/journal.pone.0242066)
Supplement: S2 File — (PDF) [file pone.0242066.s014.pdf]

MLC Foundation

Postbus 11500
2502 AM Den Haag

Dagelijkse Groenmarkt 2
2513 AL Den Haag

Tel: 31 (0)70 358 97 72

contact@mlcfoundation.org
www.mlcfoundation.org

IBAN: NL20 INGB 0007 6408 35
BIC: INGBNL2A

Chamber of Commerce: 67277012
VAT-nr: NL856908733B01

To whom it may concern

Date: May 1 2020

Dossier: Prescan study

The question has been raised why the study reported in "Health Outcomes and Experiences of Direct-to-Consumer High-Intensity Screening for Cancer and Cardiovascular Diseases" had not been first authorized by an ethical review board (IRB).

The Dutch statutory system for ethical review of medical research is regulated in the Medical research involving human subjects Act. That Act has a limited remit. The criteria for research to fall under the Act are:

a. It concerns medical research;
b. The subjects are subjected to procedures or are required to follow rules of behaviour

Research involving only questionnaires generally do not fall under the Act. This would be different if the questionnaires would be repetitive or intrusive. The Dutch system is clearly explained on the site of the (Dutch) Central Committee on Research Involving Human Subjects

Given the fact that only two questionnaires were sent out to the non referred cases and one questionnaire plus a telephone interview for the referred cases, this research would not even fall into the 'grey area' as described on the site of the Central Committee. Hence, according to Dutch law, ethical vetting of the research was not necessary.

I should add the following.
Sometimes a proposed study is forwarded to an IRB according to Act on medical research involving human subjects, simply to get a statement that the study does

not fall within the remit of mentioned Act. That statement or declaration does not mean that the IRB has vetted the study for compliance with ethical or data protection compliance.

Many research institutions have instituted not officially recognised IRB's to vet and authorise research which does not fall within the remit of the Act on medical research involving human subjects. However, this is a fairly new development and did not exist for the present study when it was initiated.

Additionally, these 'in house' IRB's follow different procedures and sometimes vet the protocol according to different criteria, as follows from a study in which we participated.[1] Regretfully that study is only available in Dutch. The study shows an appeal for legislative change. However, that change might take a while.

I may hope that this letter helps to explain why the study was not subject to IRB approval before it was initiated.

Sincerely yours

Evert-Ben van Veen Ll.M.

---

[1] https://www.medlaw.nl/nieuws/ethische-toetsing-van-niet-wmo-plichtig-onderzoek-tijd-en-
